# Key Factors Influencing Rapid Development of Potentially Dune-Stabilizing Moss-Dominated Crusts

**DOI:** 10.1371/journal.pone.0134447

**Published:** 2015-07-31

**Authors:** Chongfeng Bu, Kankan Zhang, Chunyun Zhang, Shufang Wu

**Affiliations:** 1 Institute of Soil and Water Conservation, Northwest A&F University, Yangling, Shaanxi, 712100, China; 2 Institute of Soil and Water Conservation, Chinese Academy of Sciences and Ministry of Water Resources, Yangling, Shaanxi, 712100, China; 3 College of Water Resources and Architectural Engineering, Northwest A & F University, Yangling, Shaanxi, China; University of Vigo, SPAIN

## Abstract

Biological soil crusts (BSCs) are a widespread photosynthetic ground cover in arid and semiarid areas. They have many positive ecological functions, such as increasing soil stability, and reducing water and wind erosion. Using artificial technology to achieve the rapid development of BSCs is expected to become a low-cost and highly beneficial ecological restoration measure. In the present study, typical moss-dominated crusts in a region characterized by mobile dunes (Mu Us Sandland, China) were collected, and a 40-day cultivation experiment was performed to investigate key factors, including watering frequency, light intensity and a nutrient addition, which affect the rapid development of moss crusts and their optimal combination. The results demonstrated that watering frequency and illumination had a significant positive effect (P=0.049, three-factor ANOVA) and a highly significant, complicated effect (P=0.000, three-factor ANOVA), respectively, on the plant density of bryophytes, and a highly significant positive effect on the chlorophyll *a* and exopolysaccharide contents (P=0.000, P=0.000; P=0.000, P=0.000; one-way ANOVA). Knop nutrient solution did not have a significant positive but rather negative effect on the promotion of moss-dominated crust development (P=0.270, three-factor ANOVA). Moss-dominated crusts treated with the combination of moderate-intensity light (6,000 lx) + high watering frequency (1 watering/2 days) - Knop had the highest moss plant densities, while the treatment with high-intensity light (12,000 lx) + high watering frequency (1 watering/2 days) + Knop nutrient solution had higher chlorophyll *a* contents than that under other treatments. It is entirely feasible to achieve the rapid development of moss crusts under laboratory conditions by regulating key factors and creating the right environment. Future applications may seek to use cultured bryophytes to control erosion in vulnerable areas with urgent needs.

## Introduction

Biological soil crusts (BSCs) are ecological pioneers and play very important ecological functions in the desert ecosystem. Because of their drought and cold resistance, they are widely distributed in arid and semi-arid regions. They can fix sands by aggregating surface soil [1,2], change soil nutrient cycling [3], accumulate soil nutrients [4,5], improve the soil micro-structure [6], and lay the foundation for ecosystem development [7,8]. BSCs can be divided into cyanobacteria crusts, lichen crusts, and moss crusts according to the dominating species [9], and the successional order is cyanobacteria crusts, followed by lichen crusts and moss crusts [10], with moss crusts being the advanced stage under favorable site conditions [11,12]. Mosses are poikilohydric plants that are physiologically drought tolerant. During drought, mosses lose water and become dry, and when the soil water and air humidity increases, they rapidly absorb moisture to restore normal physiological metabolic activities. Moss crusts facilitate the colonization and growth of vascular plants in some arid ecosystems due to their soil stabilization and fertility effects [12]. Moss crusts can fix carbon (C) through photosynthesis [13], increase the organic matter content in soils [14], fix sands [15], provide better fertility for the subsequent germination of vascular plant seeds, and engineer a solid foundation for the establishment of vascular plants [3,16].

Although moss crusts have a significant positive impact on the ecosystems in arid areas, their growth can take several years or even decades under natural conditions [17]. Meanwhile, mosses are very sensitive to disturbance [18], and thus, damage to stable developed mosses can easily result in changes in their coverage, composition, and functions, leading to severe soil erosion. In addition, mosses take a long time to fully recover after disturbance [19, 20], so it would be both effective and practical to cultivate artificially moss crusts for use in ecological restoration.

Compared with vascular plant restoration materials, moss crusts are better adapted to environments with drought or poor soils, especially in some of the ecosystems most vulnerable to degradation. Artificial moss crusts could immediately play a role in preventing erosion of damaged surfaces in urgent need of restoration. Technology development to accomplish fast cultivation is the first step toward this goal. Therefore, the identification of key factors that affect the rapid development of moss crusts and aid in the achievement of rapid recovery is of great practical significance for exploiting their positive ecological functions. A previous report [21] suggested that the recovery of the mosses on scalped surfaces was relatively fast even under the extreme arid conditions of the Negev Desert (with long-term annual precipitation <100 mm), however, there are few studies on the artificial cultivation of moss crusts in arid areas and their applications for ecological restoration [22,23], and most studies on mosses have concentrated on cultivating their tissues, primarily for applications in medicine [24, 25].

The feasibility of artificially establishing moss-dominated crusts needs to be examined to narrow the information gap between research and practical applications. Typical dune-stabilizing moss crusts in the Mu Us Sandy land were collected for the present experiment, and a moss crust cultivation experiment was performed using incubators that allowed for the careful control of moisture, illumination, nutrients, and other environmental factors. The key factors that affect the rapid development of moss crusts were investigated by measuring and analyzing various indicators, including plant height, plant density, and chlorophyll *a* and exopolysaccharide contents, to determine the optimal environment for the rapid development of moss crusts under constant light intensity and constant temperature and, thus, provide baseline references for engineering applications.

## Materials and Methods

### Sample collection and experimental design

Under natural conditions, asexual reproduction through fragments of stems and leaves is the primary means of moss propagation and development of moss crusts [24,25]. Based on this, typical dune-stabilizing moss-dominated (*Bryum argenteum* was the dominant species) crust samples were collected from the southeast edge of the Mu Us Sandland (Gechougou of Shenmu County, Shaanxi, 38° 53'57"N, 109° 52'44"E) administered by Northwest A&F University. Samples were brought indoors, soaked in water, and seived to remove most impurities including soil and litter. The leaf and stem fragments of moss with some cyanobacteria were ground using plant grinder after the samples were dried at 35°C. A plate-like container (diameter: 18 cm, height: 2 cm) was used as the cultivation vessel. The underlying sands at the sampling sites were placed in each container as the substrate (1.5 cm thick) (soil nutrient contents are shown in Table 1). Substrate sands and crusts layer were watered to a gravimetric water content of 13.9%, which not only completely hydrated the crusts but avoided water ponding (as determined in preliminary testing). Then 2.48 g of moss fragments were evenly sowed to achieve the ideal coverage and homogeneity (after attempting various methods, we found that manual sowing achieved the best uniformity) and cultivated in an incubator for 40 days. In general, 5–25°C is the adaptive range for most moss growth [22]. Our previous study showed that moss crusts (*Bryum argenteum* dominated) developed best in terms of plant density and height at 15°C, compared to 25°C or 35°C [26]. Similar studies demonstrated that when the temperature is higher than 17°C, moss crusts (*Barbula vinealis* dominated) coverage and moss plant growth are inhibited [24]. Accordingly, the cultivation temperature of the experiment was set to 15°C. The experimental factors and their levels were as follows: light intensity: 2,000 lx, 6,000 lx, or 12,000 lx [22,25]; watering frequency [27]: once every 2 days or once every 6 days. All treatments underwent a natural evaporative process in the growth chamber with the relative air humidity of 80%, and were supplemented every 2 days or 6 days with just enough water to bring the soil water content back to 13.9% (determined by weighing); Knop nutrient solution [22]: solution was applied by spraying once every 2 days [28] and 38 ml per container was applied each time, and the control is the same 38 ml/container of water without nutrients. There were a total of 12 treatments (shown in Table 2) and 3 replicates per treatment, for a total of 36 experimental units. This study was approved by the Institute of Soil and Water Conservation, Northwest A&F University.

**Table 1:** Soil nutrient content.

Organic N(g/kg)	Total N(g/kg)	Ammonium N (mg/kg)	Nitrate N (mg/kg)	Total P(g/kg)	Available P(mg/kg)	Total K(g/kg)	Available K(mg/kg)	pH value
6.49±0.02	0.16±0.01	4.02±0.06	3.33±0.02	0.35±0.02	4.04±0.04	25.24±0.36	109.02±0.52	8.04±0.04

**Table 2:** The design scheme for each treatment.

Treatments	Light intensity	Watering frequency	Culture medium
1	2,000 lx	1 watering / 2 days	-Knop
2	2,000 lx	1 watering / 2 days	+Knop
3	2,000 lx	1 watering / 6 days	-Knop
4	2,000 lx	1 watering / 6 days	+Knop
5	6,000 lx	1 watering / 2 days	-Knop
6	6,000 lx	1 watering / 2 days	+Knop
7	6,000 lx	1 watering / 6 days	-Knop
8	6,000 lx	1 watering / 6 days	+Knop
9	12,000 lx	1 watering / 2 days	-Knop
10	12,000 lx	1 watering / 2 days	+Knop
11	12,000 lx	1 watering / 6 days	-Knop
12	12,000 lx	1 watering / 6 days	+Knop

### Measured indicators and measuring methods

For plant density determination, 5 sampling points for each replicate plate were measured every 10 days and the values (total 3 plates) averaged. At the end of experiment (40 days duration), 4 points per plate were sampled and pooled to determine Chlorophyll *a* and exopolysaccharide contents for each replicate plate. The sampler was a cylindrical hollow tube with an inner diameter of 1.86 cm with an area of 2.72 cm^2^ (equivalent to 12.8% of the total surface area of one plate). The sample thickness was 3mm. Chlorophyll *a* and exopolysaccharide were used as indicators of the photoautotrophic activity of the biocrust organisms (moss or cyanobacteria) [29] and conditions for moss-dominated crusts development [30]. Chlorophyll *a* was extracted with acetone [31]. The specific steps for chlorophyll *a* extraction were as follows: 5 ml of 80% acetone was added to the moss samples, the samples were ground, and then placed into test tubes and left in the dark for 18 h. The sample volume was adjusted to 25 ml using 80% acetone, and then the absorbance at 645 nm (A645) and 663 nm (A663) was measured separately. The equation, chlorophyll a = 12.72 (A663)- 2.59 (A645), was used to calculate the chlorophyll *a* contents.

Exopolysaccharides, the primary components of extracellular polymers that cement soil particles and increase the erosion resistance of soils, can adhere to clay minerals by forming hydrogen bonds. The anthrone method was used to determine the exopolysaccharide content [31]: moss-dominated crusts sample collected was put into the test tube, 5 ml dilute sulphuric acid was added and then shocked for 1 h and filtered to obtain extracted sample. 1 ml extracted sample was placed in a test tube, and 5 ml water was added; the extraction took 30 min in an 80°C water bath. 0.5 ml of anthrone reagent and 5 ml of concentrated sulfuric acid were added, and the sample was kept in the 80°C water bath for another 10 min. After cooling, the absorbance was measured at 630 nm. The standard curve was plotted using a sucrose solution.

### Data analysis

The response data collected were organized and calculated using Excel 2007. One-way ANOVA and three-factor ANOVA were performed using SPSS 13.0 to examine moss density, chlorophyll *a* or exopolysaccharides responses to light intensity, watering frequency, and nutrient solution. The Duncan's multiple range test using DPS 7.05 was used for post hoc comparison of the means.

## Results

### Effects of three factors on moss growth

**Fig 1:**
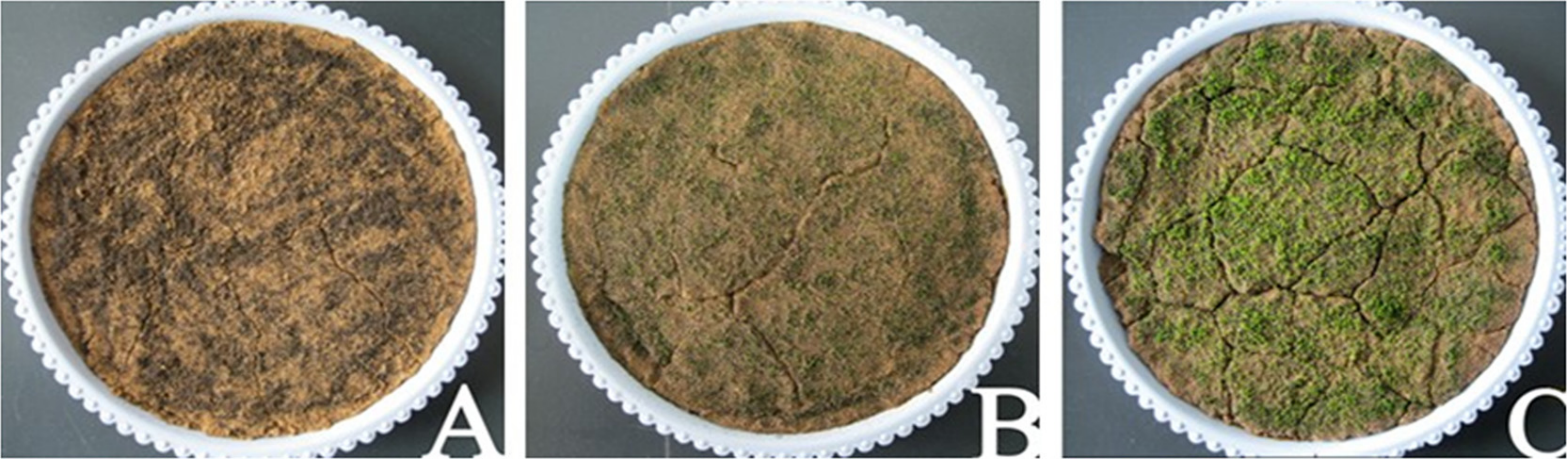
Changes to the surface of moss crust under treatment 6,000 lx+1 watering/2 days-Knop during cultivation. A, B, and C, represent the status at day 0, day 20, and day 40, respectively.

**Fig 2:**
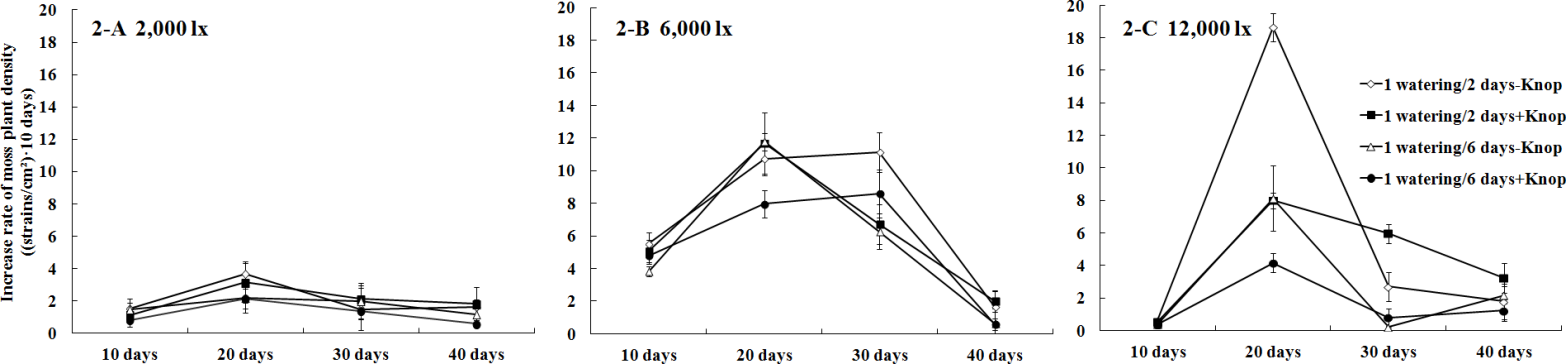
Dynamic characteristics of the growth rates of moss plant densities for four treatments under 2000 lx, 6000 lx, and 12000 lx illumination intensities, respectively. The values were presented as the Mean ± SE.

Fig 1 shows the growth status of mosses that received 6,000 lx illumination+1 watering/2 days-Knop at 0 days (A), 20 days (B) and 40 days (C). There were significant changes in the growth of the bryophytes. At the end of experiment the associated cyanobacteria could be seen. The growth rate of moss plant densities (Fig 2A, 2B and 2C) demonstrated a consistent trend under various treatments: initially fast and subsequently slow. The same trend was also observed for plant height, and the average plant height of all treatments reached 0.49±0.06 mm after 40 days. The order of the plant height under the 3 light intensities was 6,000 lx > 12,000 lx > 2,000 lx, the average plant height was 0.52±0.06 mm, 0.48±0.08 mm and 0.47±0.02 mm, respectively. Differences between them were non-significant (p>0.05).

Three-factor ANOVA (Table A in S1 File) showed that both illumination and watering frequency had significant effects on the moss plant density (P<0.05). The nutrient solution and the interaction between the 3 factors showed no significant effect on the moss plant density (P>0.05).

**Fig 3:**
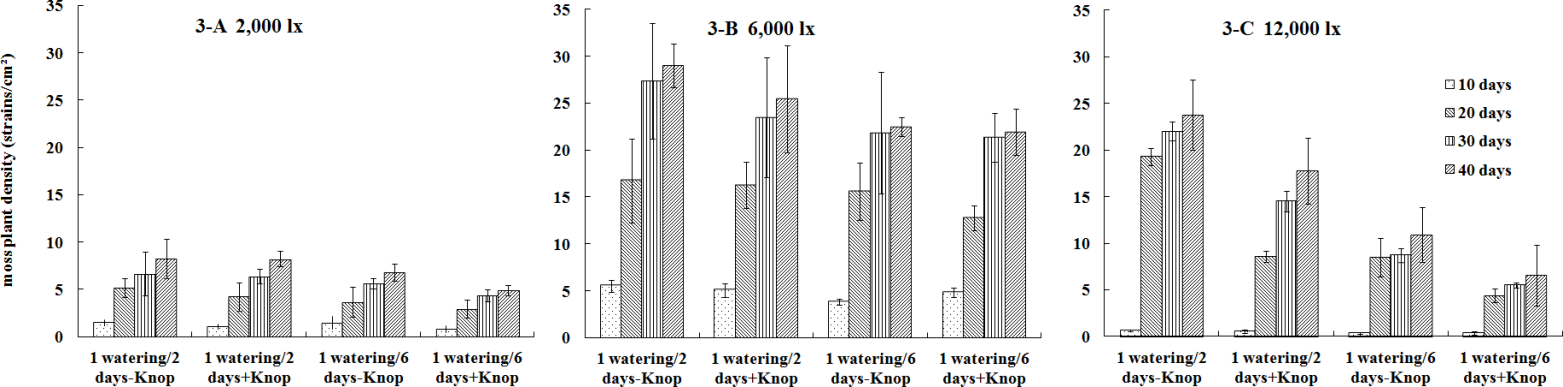
Dynamic changes in moss plant densities for four treatments under 2000 lx, 6000 lx, and 12000 lx illumination intensities, respectively. The values were presented as the Mean ± SE.

Fig 3 shows the dynamic characteristics of moss plant densities under the 3 different light intensities. Under low light conditions (2,000 lx, Fig 3A), the mosses that were watered every 2 days had higher plant densities than those that were watered every 6 days. Using the same watering frequency, mosses treated with extra water had higher plant densities than those treated with the nutrient solution, indicating that applying the nutrient solution did not promote the growth of bryophytes. Under moderate light intensity (6,000 lx, Fig 3B), the watering frequency had similar effects on moss plant densities as were observed under 2,000 lx. Nevertheless, there was only a small difference between the two watering frequencies at day 40. Under high light intensity (12,000 lx, Fig 3C), the effects of the watering frequency were similar to those observed under 2,000 lx and 6,000 lx. However, the plant density differences among the various treatments were the largest at day 40.

Overall, the order of plant density under different watering treatments, from high to low, was the same regardless of light intensity: 1 watering/2 days-Knop > 1 watering/2 days+Knop > 1 watering/6 days-Knop > 1 watering/6 days+Knop. The order of plant density for the same watering treatment but different light intensities was, from high to low, 6,000 lx > 12,000 lx > 2,000 lx. For the optimal moss plant density, the best combination of factors was 6,000 lx illumination+1 watering/2 days-Knop.

### Effects of three factors on the chlorophyll *a* of moss-dominated crusts

Under low light intensity (2,000 lx), the amount of moss was small, and there was not enough biomass to determine the chlorophyll *a* and exopolysaccharide contents. Therefore, only changes in the chlorophyll *a* of the mosses that were grown under 6,000 lx and 12,000 lx were analyzed.

Three-factor ANOVA (Table B in S1 File) demonstrated that illumination, watering frequency, watering frequency × nutrient solution and illumination × watering frequency × nutrient solution all had highly significant effects on the chlorophyll *a* contents of moss-dominated crusts (P<0.01). Illumination × nutrient solution had a significant effect (P<0.05), the effect of the nutrient solution and illumination × watering frequency was not significant (P>0.05).

**Fig 4:**
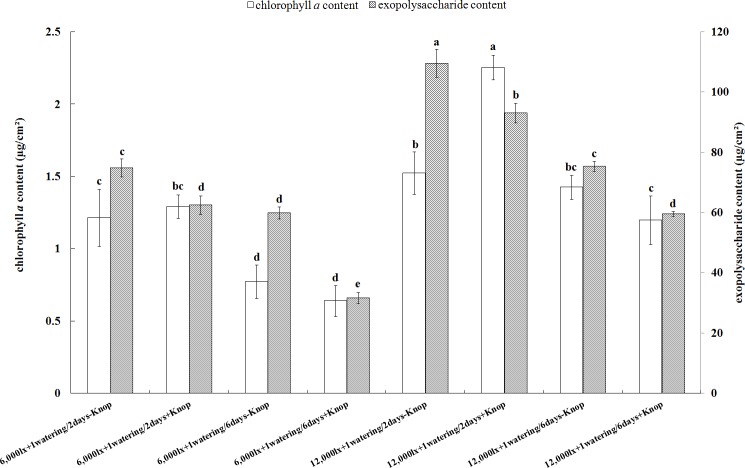
Chlorophyll *a* contents and Exopolysaccharide contents of mosses under various treatments. The values were presented as the Mean ± SE, different small letters indicate significant differences among homogeneous treatments at P < 0.05.

Fig 4 shows the effect of illumination on chlorophyll *a* contents, and the comparison of the means using Duncan’s test. The chlorophyll *a* contents of moss-dominated crusts under varying watering treatments and 12,000 lx illumination were greater than those under 6,000 lx illumination though with lower moss plant density (except for the treatment of 12,000 lx illumination+1 watering/6 days+Knop). The chlorophyll *a* contents were higher in moss-dominated crusts that were watered once every 2 days than those that were watered once every 6 days. Under 6,000 lx illumination, there were no significant differences in chlorophyll *a* contents between moss-dominated crusts treated with water and nutrient solution, regardless of watering frequency. However, a significant effect was observed for watering frequency, with greater chlorophyll *a* levels in moss-dominated crusts under the low watering frequency. In particular, chlorophyll *a* of moss-dominated crusts under the treatment 12,000 lx+1 watering/2 days+Knop was significantly larger than those of moss-dominated crusts under various treatments receiving 6,000 lx illumination, as well as those of moss-dominated crusts receiving other treatments under 12,000 lx illumination. One-way ANOVA revealed that the watering frequency significantly affected the chlorophyll *a* contents of moss-dominated crusts under 12,000 lx illumination (F = 9.883, P = 0.010), while the nutrient solution did not show a significant effect (F = 1.057, P = 0.328). Under 6,000 lx illumination, the chlorophyll *a* contents of moss-dominated crusts that were watered once every 2 days was significantly higher than those that were watered once every 6 days (F = 50.93, P = 0.000). At the high watering frequency, the chlorophyll *a* contents were greater in moss-dominated crusts treated with the nutrient solution than in those treated with extra water; and at the low watering frequency, the results were the opposite; however, these differences were not significant (F = 0.022, P = 0.884).

The order of the chlorophyll *a* content of moss-dominated crusts under different treatments was shown in Fig 4. The chlorophyll *a* contents were significantly higher in moss-dominated crusts under the 12,000 lx+1 watering/2 days+Knop treatment than in those under any other treatment. In summary, the best environmental combinations for the best development of moss-dominated crusts crust were 12,000 lx illumination+1 watering/2 days+Knop and 12,000 lx illumination+1 watering/2 days-Knop.

### Effects of three factors on the exopolysaccharide of moss-dominated crusts

Three-factor ANOVA (Table C in S1 File) showed, except for illumination × nutrient solution, that all of the other individual factors and their interactions significantly affected the exopolysaccharide contents of mosses (P<0.05).

Similar to the changes observed in the chlorophyll *a*, the effect of illumination on the exopolysaccharide contents was highly significant. Fig 4 shows that the exopolysaccharide contents of moss-dominated crusts under various treatments at 12,000 lx illumination were greater than those under 6,000 lx illumination (except for the 12,000 lx illumination+1 watering/6 days+Knop treatment), that the exopolysaccharide contents were significantly greater in moss-dominated crusts watered once every 2 days than in those watered once every 6 days, and that the exopolysaccharide contents were significantly greater in those treated with water than in those treated with nutrient solution. One-way ANOVA showed that the watering frequency significantly affected the exopolysaccharide contents of moss-dominated crusts (F = 40.343, P = 0.000), whereas the effect of the nutrient solution on their exopolysaccharide contents was not significant (F = 2.223, P = 0.167). Under 6,000 lx illumination, the exopolysaccharide content was higher for moss-dominated crusts receiving the 1 watering/2 days treatment than for those receiving the 1 watering/6 days treatment; the exopolysaccharide contents were significantly lower for moss-dominated crusts treated with the nutrient solution treatment than for those treated with water. The frequency of watering had a highly significant impact on the exopolysaccharide contents of moss-dominated crusts (F = 10.617, P = 0.009). Knop solution had a significant negative effect on the exopolysaccharide contents of moss-dominated crusts (F = 6.843, P = 0.026).

The order of exopolysaccharide contents in moss-dominated crusts under different treatments was demonstrated in Fig 4. The highest exopolysaccharide content in moss-dominated crusts was under 12,000 lx illumination+1 watering/2 days-Knop treatment. In terms of exopolysaccharide content, the best combination of factors for moss-dominated crusts crust cultivation was 12,000 lx illumination+1 watering/2 days-Knop.

## Discussion

### Factors influencing development of moss-dominated crusts

The moss species tested, *Bryum argenteum*, has hygrophilous properties and requires a certain amount of humidity or water to perform photosynthesis, respiration, and physiological metabolism. The present study demonstrated that moss-dominated crusts receiving a high watering frequency resulted in higher moss plant densities, plant heights, chlorophyll *a* contents, and exopolysaccharide contents. Mosses watered once every 2 days grew better than those watered once every 6 days, indicating that water or moisture is a key factor for the growth of bryophytes. This result is supported by related studies. For example, Kidron et al. [32,33,34] found that wetness duration of soil surface had a high positive correlation with moss growth, rather than dust-driven nutrients, direct rain precipitation, or dew. Severe desiccation of soil surface negatively affected the shoots biomass of moss crusts in Mojave Desert [35]. Other reports demonstrated that relative air humidity is a major factor limiting the growth of bryophytes and high humidity is conducive to generating more protonema [24], the optimum moisture content for the photosynthesis of mosses was 80%- 100% [36].

Mosses are primarily shade plants but can also adapt to a wide range of light intensities [36]. Of the three light intensities tested in the present experiment, moss-dominated crusts did not grow adequately under the lowest light intensity (2,000 lx), and the chlorophyll *a* and exopolysaccharide contents were the highest under 12,000 lx illumination. The possible reasons are as follows: a light intensity of 2,000 lx is not adequate for moss growth, resulting in the slow growth of the bryophytes; illumination at 12,000 lx provided relatively sufficient light, and would obviously result in development of accompanying cyanobacteria and moss plant density. These results suggest that the light inhibition point of mosses was not reached at 12,000 lx. This result is also supported by similar studies: for moss crusts, the light saturation point is approximately 1,000 μmol m^-2^ s^-1^ (approximately 80,000 lx) [36]. Moss plants under low light (2,000 lx) were significantly thinner than those under other illuminations. The higher moss plant density and lower chlorophyll *a* and exopolysaccharide content under 6,000 lx than that under 12,000 lx were probably because the interaction of 6,000 lx with the temperature and soil water content resulted in higher emergence of moss. Under 12,000 lx, the photosynthesis rate and accompanying cyanobacteria is higher resulting in higher chlorophyll *a* and exopolysaccharide content.

In theory, applying nutrient solution should increase the growth rate of bryophytes. However, treatment with Knop nutrient solution did not demonstrate any significant advantage and instead demonstrated disadvantages for plant density and plant height. This was the opposite of expectations and of prior work [24,27]. The possible reasons are as follows: the moss growth had a higher demand for water than for nutrients during the early stage of the present study, but as time extended, nutrients gradually played more important role. For example, the treatment of 12,000 lx illumination+1 watering/2 days+Knop produced a higher i rate of increase in plant density (Fig 2C) and a larger chlorophyll *a* content (Fig 4) than that without Knop. This indicates that the application of the nutrient solution would be beneficial to the growth of mosses in the long run, which is consistent with the results reported by Maestre et al. [27]. In addition, the chlorophyll *a* results of indicated that a moderate increase in the light intensity can promote moss-dominated crusts growth and that the magnesium (Mg) and N in the Knop solution can promote the synthesis of chlorophyll *a* under the high watering frequency. The exopolysaccharide contents were significantly lower in treatments with the nutrient solution than in those treated with water alone. These results may have occurred because the metal ions contained in the Knop nutrient solution inhibited the physiological activities of mosses and accompanying cyanobacteria during the growth stage, preventing moss-dominated crusts from showing significant nutrient effects [22].

As a whole, Knop solution application played a lesser role in comparison to light intensity and watering frequency in this study. However this does not imply that Knop nutrient would not be important in moss-dominated cultivation. For example, Ahmed et al. [37], proposed that the concentration of Knop solution affects the bud induction of mosses. Modified Knop medium is able to facilitate the elongation of the protonemata of *Rhodobryum giganteum* moss and prolong their growth time [38], but half-strength Knop did not clearly promote the growth of moss *Brachymenium capitulation* [39]. In our experiment, exopolysaccharide with Knop solution under higher watering frequency are higher than that without Knop, which supported its rather complicated interactive effects in combination with other factors. Therefore, the reasons underlying the somewhat unexpected results can only be speculative. Further studies are needed to determine how and why moss-dominated crusts may, or may not, respond to nutrient amendments.

### Potential applications of artificial moss crusts

Although bio-crusts are distributed widely in arid and semi-arid regions and have a large number of important ecological functions, their natural development or restoration after disturbance generally takes from several years to decades. Therefore, in recent years, researchers [24,25,26] have attempted to achieve fast cultivation of biocrusts to restore functions in the ecosystem. Especially, in many extreme conditions, bio-crusts can act as pioneers. For example, there are 76810 large-scale construction projects covering 552.8×10^4^ hm^2^ from 2001–2005 in China, which resulted in vast area of ecologically damaged lands in need of restoration. Compared to traditional tree and grass planting, artificial cultivated biocrusts is thought to have potential as a high- benefit and low-cost way to prevent erosion in these damaged surfaces that are in urgent need of stabilization. Our current results could provide some useful baseline information for selecting factors including light intensity, watering, and nutrients, for artificial cultivation of biocrusts in the lab. However, there are at least three further steps before practical applications occur in the field.

First, more factors such as air humidity and plant growth regulators, which might influence the development of moss crusts should be investigated in further studies, to enable specifying the optimal environment for fast cultivation in the lab under controlled environmental conditions. Also, the influence of substrate conditions including soil type, soil texture, and chemical properties which may affect the growth of moss crusts should be addressed. Second, cultivation of moss-dominated crusts in large pieces [40] needs to be investigated. This would determine whether it is possible to transport moss crusts from laboratory to the field and whether artificially produced moss crusts could be transplanted in the field in a similar manner as laying sod. Third, the evaluation of stress resistance and appropriate management for artificially moss-dominated crusts are essential to make them better adapted under natural conditions. In this study, we used the fragments of stem and leaf as initial moss propagules. The more elaborate method (spore propagation) for in vitro cultivation of bryophytes proposed by Duckett et al. [22] will be attempted in future studies.

Artificially-produced moss-dominated crusts are suitable for application in construction scarred sites especially those that are vulnerable to erosion. In some cases, *in situ* moss crusts cultivation, through direct spreading of moss-dominated crust samples salvaged from construction sites, will be a better choice. The cultivation and management or the protection of moss-dominated crusts under these unpredictable environments is also an important research topic for their rapid restoration. In conclusion, our current study improves the understanding of the factors influencing moss-dominated crusts development, and these results provide a basic and helpful reference for advancement from laboratory experiment to field application. Nevertheless, there is still much work to be done to narrow the information gaps and to accelerate the transition between research and practical application, for restoring and engineering moss crusts to control erosion for ecologically impaired areas in urgent need of restoration.

## Supporting Information

S1 File Table A: Variance analysis of different factors on moss plant density. Table B: Variance analysis of different factors on moss chlorophyll *a* content. Table C: Variance analysis of different factors on moss exopolysaccharide content.
(DOCX)
