# Supplementary material for: Key Factors Influencing Rapid Development of Potentially Dune-Stabilizing Moss-Dominated Crusts
Source: PLoS One. 2015 Jul 31;10(7):e0134447. doi: 10.1371/journal.pone.0134447 (PMC4521833; doi:10.1371/journal.pone.0134447)
Supplement: S1 File — Table A: Variance analysis of different factors on moss plant density. Table B: Variance analysis of different factors on moss chlorophyll a content. Table C: Variance analysis of different factors on moss exopolysaccharide content. (DOCX) [file pone.0134447.s001.docx]

**Table A Variance analysis of different factors on moss plant density**

| **factor** | **SS** | **df** | **MS** | **F** | **P** |
| --- | --- | --- | --- | --- | --- |
| illumination | 1285.892 | 2 | 642.946 | 13.531 | 0.000 |
| watering frequency | 196.391 | 1 | 196.391 | 4.133 | 0.049 |
| culture medium | 59.609 | 1 | 59.609 | 1.254 | 0.270 |
| illumination×watering frequency | 85.972 | 2 | 42.986 | 0.905 | 0.414 |
| illumination × culture medium | 31.868 | 2 | 15.934 | 0.335 | 0.717 |
| watering frequency × culture medium | 4.852 | 1 | 4.852 | 0.102 | 0.751 |
| illumination × watering frequency × culture medium | 7.632 | 2 | 3.816 | 0.080 | 0.923 |

**Table B Variance analysis of different factors on moss chlorophyll *a* content**

| **factor** | **SS** | **df** | **MS** | **F** | **P** |
| --- | --- | --- | --- | --- | --- |
| illumination | 2.313 | 1 | 2.313 | 137.595 | 0.000 |
| watering frequency | 1.894 | 1 | 1.894 | 112.656 | 0.000 |
| culture medium | 0.075 | 1 | 0.075 | 4.487 | 0.050 |
| illumination×watering frequency | 0.001 | 1 | 0.001 | 0.072 | 0.792 |
| illumination × culture medium | 0.118 | 1 | 0.118 | 7.038 | 0.017 |
| watering frequency × culture medium | 0.512 | 1 | 0.512 | 30.475 | 0.000 |
| illumination × watering frequency × culture medium | 0.209 | 1 | 0.209 | 12.412 | 0.003 |

**Table C Variance analysis of different factors on moss exopolysaccharide content**

| **factor** | **SS** | **df** | **MS** | **F** | **P** |
| --- | --- | --- | --- | --- | --- |
| illumination | 4431.527 | 1 | 4431.527 | 577.907 | 0.000 |
| watering frequency | 4827.311 | 1 | 4827.311 | 629.520 | 0.000 |
| nutrient solution | 1993.647 | 1 | 1993.647 | 259.988 | 0.000 |
| illumination×watering frequency | 179.117 | 1 | 179.117 | 23.358 | 0.000 |
| illumination×nutrient solution | 26.800 | 1 | 26.800 | 3.495 | 0.080 |
| watering frequency × nutrient solution | 85.928 | 1 | 85.928 | 11.206 | 0.004 |
| illumination×watering frequency×nutrient solution | 103.878 | 1 | 103.878 | 13.547 | 0.002 |
